# Bilateral Middle Cerebellar Peduncle Infarction Presenting With Vertigo and Hearing Impairment Mimicking Peripheral Vestibulopathy

**DOI:** 10.7759/cureus.75623

**Published:** 2024-12-12

**Authors:** Nur Amalina Ramli, Sanihah Abdul Halim, Mohd Ezane Aziz

**Affiliations:** 1 Department of Internal Medicine (Neurology), School of Medical Sciences, Universiti Sains Malaysia, Kubang Kerian, MYS; 2 Brain and Behaviour Cluster, School of Medical Sciences, Universiti Sains Malaysia Specialist Hospital, Kubang Kerian, MYS; 3 Department of Radiology, School of Medical Sciences, Universiti Sains Malaysia, Kubang Kerian, MYS

**Keywords:** acute hearing loss, aica infarct, middle cerebellar peduncle, stroke, vertigo

## Abstract

Bilateral middle cerebellar peduncle (MCP) infarction is a rare manifestation of ischemic stroke. We report a middle-aged male patient who presented with acute onset of vertigo, left ear deafness, and severe ataxia. Magnetic resonance imaging (MRI) of the brain confirmed the presence of infarction in the bilateral middle cerebellar peduncles due to stenosis of the posterior circulation arteries. This case highlights the importance of recognizing this clinical syndrome, as the symptoms may resemble those of peripheral vestibulopathy. Timely recognition is of paramount importance, given that untreated posterior circulation stroke can result in poor neurological outcomes.

## Introduction

Bilateral middle cerebellar peduncle (MCP) infarction represents a rare neurological manifestation of acute ischemic stroke. This clinical syndrome is characterized by a sudden onset of vertigo, ataxia, and dysarthria, and it may also present with acute hearing impairment or other symptoms of brainstem infarction [1,2]. The pathophysiology is thought to arise from hypoperfusion due to chronic atherosclerosis affecting the posterior circulation [1,3,4]. The middle cerebellar peduncle is particularly vulnerable to ischemia due to its anatomical location at the border-zone area between the anterior inferior cerebellar artery (AICA) and superior cerebellar artery (SCA) territories [4].

## Case presentation

A 45-year-old male with a history of hypertension presented with a sudden onset of vertigo, diminished hearing in the left ear, and gait instability. He received treatment at a local hospital for hypertensive urgency with an initial diagnosis of peripheral vertigo attributed to the accompanying hearing impairment. He was discharged home after a few days of hospitalization.

However, the patient’s symptoms deteriorated, resulting in a need for assistance with activities of daily living due to significant ataxia. Additionally, he developed dysphagia with very minimal oral intake, which prompted a referral to our center for further evaluation and management.

Upon examination, the patient’s blood pressure was 160/90 mmHg. He had difficulty standing due to severe ataxia. There was bilateral horizontal nystagmus, but no weakness of the extraocular muscles. The head impulse test was negative. Evaluation of the eighth cranial nerves revealed left-sided sensory-neural hearing loss with a positive Rinne test and lateralization of sound to the unaffected, right ear during Weber test. The gag reflexes were diminished, and the patient experienced dysphagia. Other cranial nerves were intact. His speech was dysarthric, displaying characteristics of cerebellar involvement, alongside dysmetria of the left upper extremity, impaired coordination of the bilateral lower limb, and a broad-based ataxic gait. There were no signs of neuromuscular weakness or sensory deficit. Additional systemic and cardiovascular examinations yielded normal findings.

**Figure 1:**
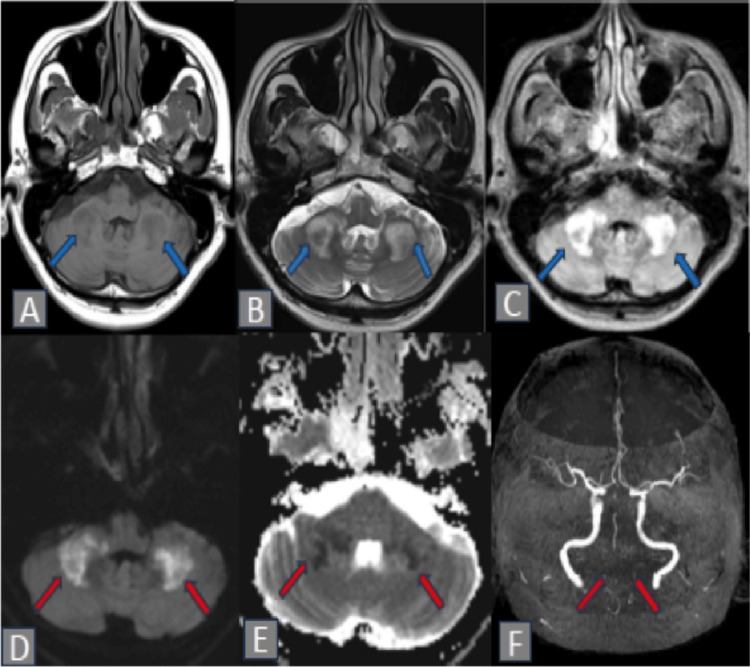
Magnetic Resonance Imaging (MRI) of the brain images of Bilateral Middle Cerebellar Peduncle Infarction Bilateral middle cerebellar peduncle infarction: (hypointensity on T1-weighted image (A), hyperintensity on both T2-weighted image (B) and fluid-attenuated inversion recovery (FLAIR) (C), and restricted diffusion on diffusion-weighted image (DWI) (D) and apparent diffusion coefficient (ADC) (E) images. Magnetic resonance angiography (MRA) shows inadequate visualization of the vertebrobasilar arteries (F), suggesting significant stenosis.

Magnetic resonance imaging (MRI) of the brain (Figure 1) revealed well-defined hyperintense lesions located at the bilateral middle cerebellar peduncles on T2-weighted and fluid-attenuated inversion recovery (FLAIR) images, accompanied by restricted diffusion observed on diffusion-weighted imaging (DWI) and apparent diffusion coefficient (ADC), indicative of acute infarction. Lacunar infarctions were also visualized within the bilateral pons. The magnetic resonance angiography (MRA) demonstrated severe stenosis of the bilateral vertebral and basilar arteries. (Figure 1F).

A 72-hour Holter electrocardiogram monitoring was conducted, which showed a heart rate ranging between 68 and 139 beats per minute without any episodes of atrial fibrillation or other arrhythmias. An echocardiogram displayed satisfactory cardiac contractility with an ejection fraction of 67% and normal-sized cardiac chambers, without evidence of a clot or thrombus. No left ventricular hypertrophy was seen. Other baseline blood tests were within normal limits: complete blood count, and renal and liver function tests. The fasting blood glucose was 4.6 mmol/L (normal value 3.9-5.5 mmol/L) and the HbA1C was 6.5% (normal value <5.5%). The fasting lipid profile showed total cholesterol of 3.67 mmol/L (normal value <5.17mmol/L), triglyceride of 1.7 mmol/L (normal value <1.7 mmol/L) and LDL-C of 2.17 mmol/L (normal value < 2.6 mmol/L). Pure tone audiometry was attempted. However, the patient had severe vertigo and was unable to complete the test.

The patient was initiated on dual antiplatelet therapy for 90 days, followed by a single antiplatelet drug, in conjunction with standard ischemic stroke care protocols. Given the delayed presentation, with established infarction beyond the time window for reperfusion therapy, the management was directed toward optimum medical treatment.

Notably, despite extensive rehabilitation efforts, the patient sustained severe ataxia and residual left-sided hearing loss after six months of therapeutic intervention.

## Discussion

Bilateral middle cerebellar peduncle infarction occurs in approximately 0.12% of ischemic stroke cases [2]. It is often associated with infarctions in other regions supplied by posterior circulation arteries, such as the pons and cerebellum [3]. The mechanisms of vascular occlusion are mainly due to large artery atherosclerosis and hypoperfusion [1,3,4].

The primary blood supply to the middle cerebellar peduncles is predominantly derived from the anterior inferior cerebellar artery (AICA), with additional contributions from the superior cerebellar artery (SCA) through anastomoses between these two vessels. The middle cerebellar peduncles are located within the watershed region between the AICA and SCA territories [4]. In our case, poorly visualized posterior circulation arteries in the brain magnetic resonance angiography (MRA) suggest a large vessel occlusion or stenosis of the vertebrobasilar arteries. The resultant hypoperfusion has led to infarction within the watershed area, affecting both middle cerebellar peduncles.

The vascular territory of the AICA also includes the lateral pons, anterior inferior cerebellum, and the inner ear [5]. Infarctions within the AICA territories are relatively uncommon, owing to extensive collateral circulation. An AICA territory infarct may present with acute hearing loss and/or tinnitus, resulting from ischemia of the labyrinthine artery, which supplies the cochlea and vestibular apparatus, as well as the facial (VII) and vestibulocochlear (VIII) cranial nerves [5]. Lee et al. reported sensorineural hearing loss in 11 out of 12 patients (92%) with middle cerebellar peduncle infarction due to AICA syndrome [6].

Since the clinical presentation of acute onset of vertigo, ataxia, and hearing loss is also frequently observed in cases of peripheral vestibulopathy, such as vestibular neuritis or Meniere’s disease, the diagnostic process can be complex [7]. Certain clinical indicators may suggest a central etiology rather than peripheral vestibulopathy. These indicators include severe ataxia, persistent vertigo lasting more than 72 hours, spontaneous nystagmus, and the presence of other brainstem symptoms, particularly in individuals with preexisting stroke risk factors [8]. A comprehensive neurologic examination should be focused on additional brainstem signs such as crossed-long tract signs, gaze palsy, facial palsy, or Horner syndrome. Moreover, a complete neuro-otologic and vestibular assessment should be performed [7,8].

The management of AICA territory infarction primarily consists of antiplatelet or anticoagulation therapies following the established ischemic stroke protocols. However, given that AICA syndrome usually occurs due to thrombosis of the large arteries, particularly AICA or basilar artery, leading to watershed infarct of the bilateral middle cerebellar peduncles, early endovascular therapy may have an important role [9].

Dong et al. reported two cases of bilateral middle cerebellar peduncle infarction that demonstrated significant recovery following endovascular intervention [9]. Patients presenting with severe stenosis of the vertebrobasilar artery, if not treated promptly, face an increased risk of extensive posterior circulation infarction and pose a substantial threat to life. Evidence supporting endovascular treatment for middle cerebellar peduncle infarctions related to vertebrobasilar artery involvement remains insufficient [9,10]. However, there is an increasing body of evidence highlighting the efficacy of endovascular interventions in cases of rapidly deteriorating neurological status associated with advanced vertebrobasilar artery stenosis [10]. Without early treatment, the prognosis of patients with bilateral middle cerebellar peduncle infarction due to large artery atherosclerosis is substantially poor.

## Conclusions

Bilateral middle cerebellar peduncle infarction represents a type of watershed infarction resulting from hypoperfusion associated with large artery occlusive disease in the posterior circulation. Timely recognition of its clinical manifestation is crucial, as failure to address this condition may result in unfavorable neurological outcomes. Given that the presentation can resemble peripheral vestibulopathy, a comprehensive evaluation of brainstem involvement, alongside precise neuroimaging, is imperative. Future research addressing the challenges related to diagnostic strategies and therapeutic interventions is required in bilateral middle cerebellar peduncle infarction.
